# The special sauce of the Cancer Prevention and Control Research Network: 20 years of lessons learned in developing the evidence base, building community capacity, and translating research into practice

**DOI:** 10.1007/s10552-023-01691-1

**Published:** 2023-06-24

**Authors:** Stephanie B. Wheeler, Rebecca J. Lee, Alexa L. Young, Adam Dodd, Charlotte Ellis, Bryan J. Weiner, Kurt M. Ribisl, Prajakta Adsul, Sarah A. Birken, María E. Fernández, Peggy A. Hannon, James R. Hébert, Linda K. Ko, Aaron Seaman, Thuy Vu, Heather M. Brandt, Rebecca S. Williams

**Affiliations:** 1 Center for Health Promotion and Disease Prevention, University of North Carolina at Chapel Hill, Chapel Hill, NC USA; 2 Lineberger Comprehensive Cancer Center, University of North Carolina at Chapel Hill, Chapel Hill, NC USA; 3 Department of Health Policy and Management, Gillings School of Global Public Health, University of North Carolina at Chapel Hill, 135 Dauer Drive, CB#7411, McGavran Greenberg Hall, Chapel Hill, NC 27599-7411 USA; 4 Impact Measurement and Visualization Team, Health Sciences Library, University of North Carolina at Chapel Hill, Chapel Hill, NC USA; 5 Department of Global Health, University of Washington, Seattle, WA USA; 6 Department of Health Systems and Population Health, University of Washington, Seattle, WA USA; 7 Department of Health Behavior, Gillings School of Global Public Health, University of North Carolina at Chapel Hill, Chapel Hill, NC USA; 8 Department of Internal Medicine, University of New Mexico, Albuquerque, NM USA; 9 Cancer Control and Population Sciences Research Program, University of New Mexico Comprehensive Cancer Center, Albuquerque, NM USA; 10 Department of Implementation Science, Wake Forest University School of Medicine, Winston-Salem, NC USA; 11 Department of Health Promotion and Behavioral Sciences, School of Public Health, University of Texas Health Science Center at Houston, Houston, TX USA; 12 Health Promotion Research Center, Department of Health Systems and Population Health, University of Washington, Seattle, WA USA; 13 Cancer Prevention and Control Program, Arnold School of Public Health, University of South Carolina, Columbia, SC USA; 14 Department of Epidemiology and Biostatistics, Arnold School of Public Health, University of South Carolina, Columbia, SC USA; 15 Department of Nutrition, Connecting Health Innovations LLC, Columbia, SC USA; 16 Department of Internal Medicine, Carver College of Medicine, University of Iowa, Iowa City, IA USA; 17 Holden Comprehensive Cancer Center, University of Iowa, Iowa City, IA USA; 18 HPV Cancer Prevention Program, St. Jude Children’s Research Hospital, Memphis, TN USA; 19 Department of Epidemiology and Cancer Control, St. Jude Children’s Research Hospital, Memphis, TN USA

**Keywords:** Cancer prevention and control, Community engagement, Research translation, Implementation science, Collaboration, Mixed methods

## Abstract

**Purpose:**

The Cancer Prevention and Control Research Network (CPCRN) is a national network focused on accelerating the translation of cancer prevention and control research evidence into practice through collaborative, multicenter projects in partnership with diverse communities. From 2003 to 2022, the CPCRN included 613 members.

**Methods:**

We: (1) characterize the extent and nature of collaborations through a bibliometric analysis of 20 years of Network publications; and (2) describe key features and functions of the CPCRN as related to organizational structure, productivity, impact, and focus on health equity, partnership development, and capacity building through analysis of 22 in-depth interviews and review of Network documentation.

**Results:**

Searching Scopus for multicenter publications among the CPCRN members from their time of Network engagement yielded 1,074 collaborative publications involving two or more members. Both the overall number and content breadth of multicenter publications increased over time as the Network matured. Since 2004, members submitted 123 multicenter grant applications, of which 72 were funded (59%), totaling more than $77 million secured. Thematic analysis of interviews revealed that the CPCRN’s success—in terms of publication and grant productivity, as well as the breadth and depth of partnerships, subject matter expertise, and content area foci—is attributable to: (1) its people–the inclusion of members representing diverse content-area interests, multidisciplinary perspectives, and geographic contexts; (2) dedicated centralized structures and processes to enable and evaluate collaboration; and (3) focused attention to strategically adapting to change.

**Conclusion:**

CPCRN’s history highlights organizational, strategic, and practical lessons learned over two decades to optimize Network collaboration for enhanced collective impact in cancer prevention and control. These insights may be useful to others seeking to leverage collaborative networks to address public health problems.

## Introduction

Cancer morbidity and mortality disparities are most pronounced among rural, minoritized, low-income and uninsured sub-populations in the United States [1–3]. Indeed, it is often the preventable and “good prognosis” cancers—the ones most amenable to primary prevention, early detection and appropriate treatment—where research has shown the greatest disparities gaps in outcomes across sub-populations [4, 5]. Reasons for these disparities are complex. Fortunately, evidence-based interventions (EBIs) are available to address multilevel challenges leading to these disparities, such as: (1) lack of access to care, (2) gaps in coverage between initial screenings and diagnostic follow-up, (3) lack of awareness, (4) fear, (5) structural racism and discrimination, and (6) previous negative healthcare experiences [6, 7]. Still, implementing these EBIs in clinical settings and in public health lags the science [8–12]. To address these apparent and longstanding gaps, the Centers for Disease Control and Prevention (CDC), in conjunction with the National Cancer Institute (NCI), initiated the Cancer Prevention and Control Research Network (CPCRN). The CPCRN (previously the Cancer Research Network (CRN) during the first funding cycle) is a cancer-focused thematic research network of the Prevention Research Centers (PRC) program, the CDC’s flagship program for preventing and controlling chronic diseases and has been consistently funded by the CDC since 2002 [13].

CPCRN comprises academic, public health, clinical, organizational, and community partners who collaborate to conduct partnered research in cancer prevention and control, particularly in medically underserved groups [14]. Using a health equity lens, CPCRN’s current mission is to “accelerate the adoption, implementation, and sustainment of evidence-based cancer prevention and control strategies in communities, enhance large-scale efforts to reach and reduce the burden of cancer among medically underserved populations, deepen our understanding of the predictable processes that achieve those goals, and develop the dissemination and implementation (D&I) workforce in cancer prevention and control” [15]. The vision of the CPCRN is to reduce the burden of cancer in U.S. populations and eliminate cancer health disparities. For the past 20 years, the CPCRN members have pursued this shared mission and vision through many different mechanisms, tracked, in part, through our progress reporting system managed by the CPCRN Coordinating Center. We updated our strategic plan in 2017, and since that time, the CDC’s Science Impact Framework has guided the CPCRN activities, progress reporting and dissemination across five domains of CDC scientific influence that drive health outcomes—disseminating science (e.g., publications, presentations, grants), creating awareness (e.g., providing subject matter expertise, trainings, toolkits and other dissemination communications), catalyzing action (e.g., technology creation, testimony, presentations to policymakers), effecting change (e.g., building public health capacity, developing registries/surveillance resources, workforce development, influencing policy, clinical recommendations or public health practice change) and shaping the future (e.g., influencing implementation and sustainment of public health programs, reducing economic burden of disease) [16, 17]. By defining a concrete set of metrics for measurement and prioritization, this Impact Framework has helped prioritize the CPCRN activities in recent years, whereby members and Collaborating Centers work toward and report progress on those substantial contributions aligned with these domains of scientific influence.

**Fig. 1:**
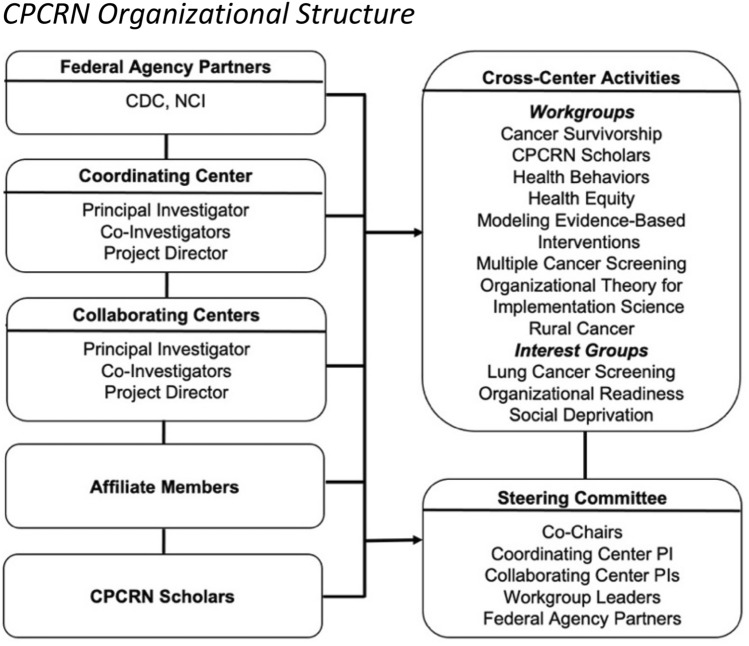
Current CPCRN organizational structure

CPCRN members have worked with national, state, and local partners to reduce cancer risk, improve routine prevention and screening and timely treatment, reduce cancer death rates, enhance the value and effectiveness of cancer care, and mitigate disparities by advancing the science and practice of D&I in cancer prevention and control [18, 19]. Our 247 current the CPCRN members conduct partnered and engaged cancer research through multicenter Workgroups and Interest Groups organized across eight currently funded Collaborating Centers and numerous community sites and Affiliate members, crossing academic disciplines and geographic boundaries. CPCRN members are supported in their work by the CPCRN Coordinating Center which has been based at the University of North Carolina at Chapel Hill since 2004, along with a robust and active internal Steering Committee and Network policies and resources that seek to clarify, simplify, and enhance collaborative productivity (Fig. 1). Key stakeholders in this work include the CDC, NCI, American Cancer Society, state Cancer Prevention and Control Branches of local Departments of Health and Human Services, academic researchers, clinical practitioners, and community organizations interested in improving cancer prevention and control implementation.

Our overarching objective in this manuscript is to clarify the ingredients of the “special sauce” of the CPCRN, that is: how has a large and complex, geographically dispersed and loosely connected network of individuals balanced a consistent mission and vision with a dynamic approach to pursuing that mission and vision over time? We attempt to resolve this question in three ways, by: (1) first, demonstrating the extent and nature of Network collaborations and expertise through a bibliometric analysis of all member publications over the past 20 years; (2) second, describing major themes that characterize the CPCRN in terms of its organization and emphasis on health equity, community partnership development, and capacity building through analysis of 22 in-depth interviews with key informants involved in the Network from inception to present; and (3) third, illustrating how strategically-developed organizational structures of the Network can meaningfully support ongoing engagement across investigators, their many institutions, and their community partners through Network policies, procedures, and norms. Our description of the longstanding the CPCRN offers lessons for population scientists across many fields who are interested in engaging with or leading formal research networks.

## Methods

### Overview

We employed a mixed methods approach using a convergent parallel design to conduct this study. Quantitative methods were used to analyze collaborative the CPCRN publications and grants; qualitative methods were used to analyze key informant interviews and existing policies and procedures documents to better understand how Network structures, processes, strategy, and culture support its people in achieving a shared purpose. Together, these methods provide a more comprehensive picture of how CPCRN pursues its mission and vision in alignment with its strategic plan and logic model, both of which have been updated as needed over time.

### Quantitative methods

From two data sources—Scopus and CPCRN’s internal progress reporting system—a retrospective, longitudinal, descriptive design was used to analyze existing bibliographic and administrative data on multicenter collaborations. To assess the growth and impact of total CPCRN publications, authorship data from Scopus were collected using researchers’ first and last name or Author ID (Scopus), as well as center affiliation, for 613 CPCRN members identified through the CPCRN membership directory and archives. Searches were filtered to focus specifically on years ranging from 2003 to 2022, as the first funding cycle effectively began in October 2002, and 3 months of this calendar year would not have been enough time to publish collaboratively. Authors’ records were limited to articles published after their respective center’s start date in the Network, or, if they were an Affiliate member, the date after which they were formally approved by the Steering Committee to participate in the Network. Duplicate and false-positive records were removed. Records for Medical Subject Heading (MeSH) term extraction were searched and downloaded on 23 September 2022. To focus on the growth of collaborative efforts within the Network, we retrieved multicenter publications, defined as such if publications involved two or more CPCRN Collaborating Center authors or Affiliate members from different institutions within CPCRN. For the bibliographic analysis, each author record was assigned a center-level identifier and linked with unique article-level identifiers to construct co-authorship clusters for each year. Custom Python scripts, R, version 3.0.2, and Excel, version 16.66.1, were used to parse, process, and summarize data, and VOSviewer, version 1.6.18, was used to construct data visualizations of the clusters [20–25].

The CPCRN Coordinating Center employs an online administrative reporting system to monitor and evaluate Network outputs and outcomes by collecting detailed information from Collaborating Centers annually about center-specific and multicenter publications, presentations, grant applications, trainings, reports/plans/policies, and other CPCRN-related activities [26]. Through the reporting system, the Coordinating Center also collects narrative data regarding requests for scientific expertise, awards and honors, and various other indicators of CPCRN impact following the CDC Impact Framework [16, 17]. For the multicenter grants analysis, the research team relied on CPCRN Progress Reporting data submitted to the Coordinating Center every 6 months until 2017, after which time the Network transitioned to annual progress reporting periods. Because grants data were not collected until 2004 and the progress reporting period for 2022 has yet to be collected and analyzed for the 2022 funding year, the research team narrowed their focus on multicenter grant applications and awards between the years of 2004 and 2021. Multicenter grant records were managed in Microsoft Excel, through which duplicates were removed and numerical discrepancies corrected; descriptive statistics summarizing numbers of applications, awards, and corresponding funding amounts were analyzed using IBM SPSS Statistics software, version 28.0 [22, 27].

### Qualitative methods

#### Key informant interviewee selection and recruitment

Semi-structured, key informant interviews were conducted among CPCRN leaders and members, including those currently involved in the Network and past members no longer involved. Authors SBW, RJL, ALY and RSW compiled a full list of all Principal Investigators (PIs), Project Directors (PDs), Federal Agency Partners and affiliates in leadership roles within the Network (e.g., Workgroup Co-Chairs), from which the team identified a diverse group of Network members and partners, past and present, who were perceived as: instrumental in shaping and/or progressing the Network; and/or had participated over the course of many years, potentially spanning multiple funding cycles and could comment on CPCRN’s evolution over time. These individuals represented diverse professional and academic disciplines, and their roles, responsibilities, and level and duration of engagement varied. A total of 49 individuals were sent initial and follow-up recruitment emails, modifying language as needed to reflect their Network role and status of involvement (i.e., currently active or formerly active). Those who expressed interest were sent additional study details, and upon providing written consent to voluntarily participate, were formally enrolled into the study. No compensation was provided.

#### Interview data collection

With a primary aim of gaining insight into members’ perceptions of the Network, unique experiences, best practices, lessons learned, and feedback/recommendations, the study team developed a semi-structured interview guide for use among participants. Informed by suggestions from co-authors, the guide went through several iterations before being finalized. The final draft was modified slightly to align with characteristic differences among participants, predominately relating to: Network role and respective responsibilities/contributions; center or agency with which they were or are currently affiliated; and extent of involvement, in terms of both participation duration and time elapsed since departing from or joining the Network. Over the 3-month data collection window, one team member (ALY) conducted a total of 22 one-on-one interviews averaging 45–50 min each. Characteristics of interviewees are described in Table 1, and the full interview guide is provided in Table 2. In advance of each session, participants received both written and verbal assurance of concrete measures that would be taken by the study team to maintain and protect confidentiality and privacy. As a result, all 22 participants (100%) provided clear, verbal consent to be recorded. The interviewer captured audio recordings via Zoom, version 5.11.0, and all interviews were transcribed with the support of Otter.ai software [28, 29]. Potential identifying information was redacted from all transcripts, and data cleaning corrected errors in transcription.

**Table 1:** *Interview Participant Characteristics*

Current Network Role	Count
Principal Investigator, *active*	6
Principal Investigator, *inactive*	2
Federal Agency Partner, *active*	6
Federal Agency Partner, *inactive*	1
Affiliate Member, *active*	3
Project Director, *active*	2
Project Director, *inactive*	1
Co-Investigator, *active*	1
Total	22

**Table 2:** Interview guide

A. Introduction & participation/engagement
∙ How and when did you become involved in the Network? – During what years and/or funding cycles have you been/were you involved? ∙ What was your original role in the Network? – How have/did your role and responsibilities change(d) over time? ∙ How do/did you feel about the intra-Network dynamic? ∙ What are/were some professional benefits to being involved
B. Network structure & operations
∙ What makes CPCRN work (i.e., its “special sauce”)? – What are some of the challenges? ∙ What sets the Network apart from other organizations?
C. Network progress & impact
∙ In what way(s) has CPCRN had meaningful impact? – What are some key indicators/metrics of impact that come to mind? – What factors have enabled CPCRN to sustain and/or enhance its impact over time? ∙ What can CPCRN do to ensure continued growth and enhanced impact in the future? ∙ To what extent has health equity been/was health equity emphasized by the Network throughout your involvement? – What has CPCRN done to center health equity in its activities? – What could CPCRN do to better center health equity moving forward?
D. Community-academic partnerships
∙ What are some examples of CPCRN projects that involved community-academic partnerships? – What kinds of activities were carried out? – What did the outputs/outcomes look like? – How were these shared back with the respective community(ies)? ∙ What has/did community participation look(ed) like in your experience (i.e., roles/responsibilities held, contributions, dynamics, etc.)? – How do/have these community-academic partnerships empower(ed) the community?
E. Capacity-building
∙ In what capacity-building activities have/were you or your Center (been) involved (e.g., trainings, mini-grants, serving as external evaluators, etc.) during your time in the Network? ∙ Who, if anyone, might you recommend we contact to learn more?
F. Closing
∙ What recommendations/lessons learned would you emphasize to other Networks who are similarly engaged in cross-Center, collaborative research? ∙ What else would you like to add about CPCRN?

#### Coding and analysis

A coding team consisting of SBW, RJL, ALY, and CE met to establish a collaborative coding approach and develop the codebook structure. They independently reviewed three different transcripts and developed an initial comprehensive collection of themes and sub-themes that emerged most prominently across the conversations. Primary themes included: (1) multidisciplinary expertise; (2) collaborative culture; (3) supportive funding; (4) Network structures and policies; (5) professional and leadership development; (6) community orientation; (7) adaptability; and (8) attention to ongoing learning and improvement. After defining and refining these codes, the codebook and all transcripts were uploaded into Dedoose qualitative data management software, Version 9.0.62 [30]. Nine additional transcripts were coded simultaneously by dual coders (ALY and CE). Based upon largely consistent code application and a shared understanding of their definitions, the team determined that there was strong intercoder reliability. As such, the final ten transcripts were then coded independently by one coder (ALY). Coded transcripts were subsequently analyzed and synthesized to identify impactful Network features and functions.

#### Policies and procedures documents review

In addition, we conducted a comprehensive review of existing CPCRN documents, including policies, procedures, and web-based resources, to enumerate and characterize the breadth and variety of written processes and structured materials, developed and modified over time with the shared goal of orienting, supporting, and continuously guiding members within the Network.

## Results

### Quantitative

#### Bibliometric literature review findings

**Fig. 2:**
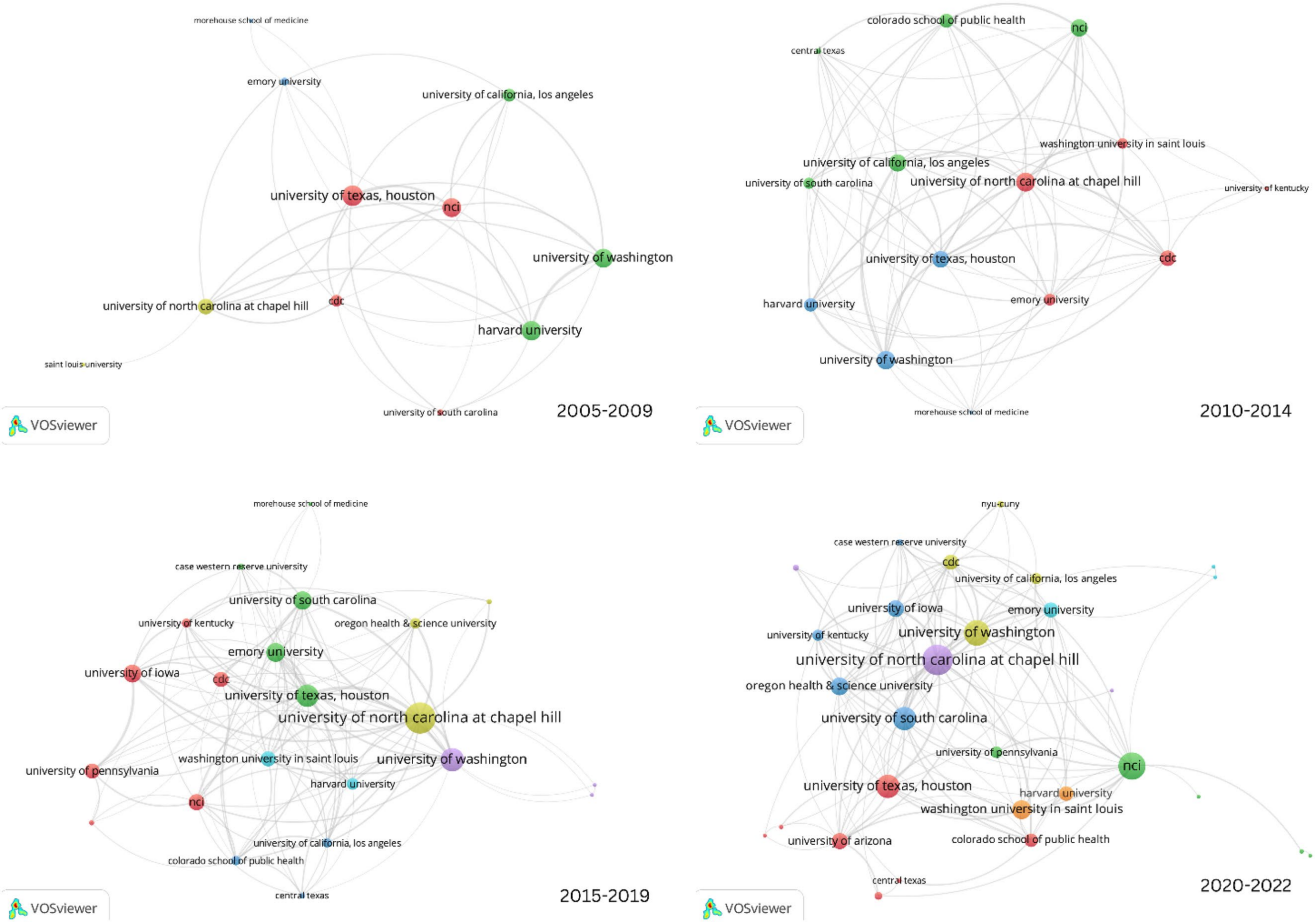
Density of multicenter publications by CPCRN Collaborating Center (2005–2022) [Cycle 1 (2002–2004) is not shown because of its shorter duration of funding; as a result, Cycle 1 is not comparable to other funding cycles]

**Fig. 3:**
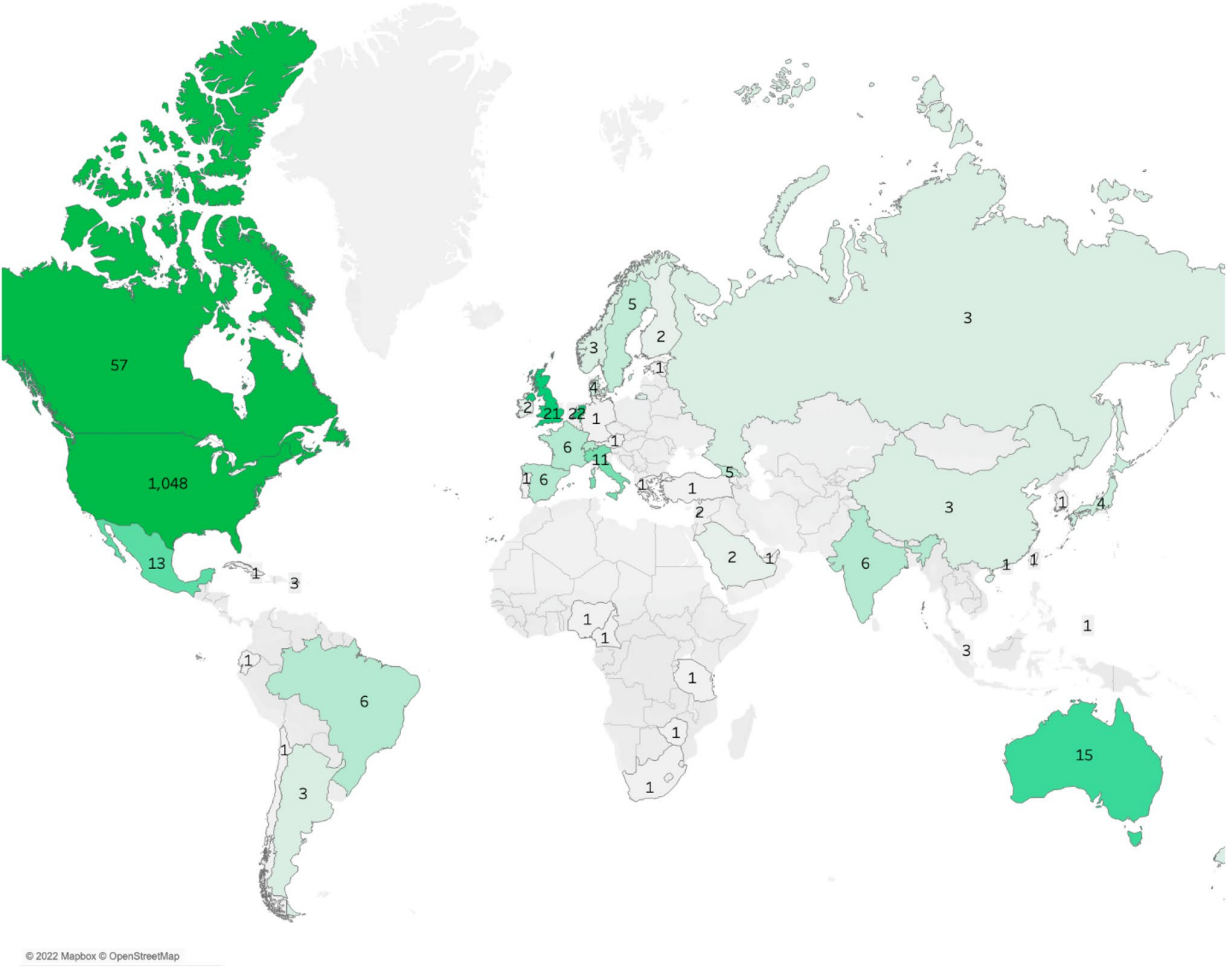
Number of the CPCRN multicenter publications by country of collaboration (2003–2022)

In the 20 years since CPCRN’s inception through the Special Interest Projects (SIP) mechanism of the CDC Prevention Research Center’s (PRC) Program, the Network has consisted of a cumulative total of 19 different Collaborating Centers, each of whom participated in one or more of the five cycles funded (Table 3). The depth and breadth of collaboration has grown over time, as shown by the increase in both the density of overall and collaborative publications across centers, as well as the number of nodes/centers in the Network over time (Fig. 2). Not only have CPCRN Collaborating Centers developed strong collaborations with each other, but they have also engaged and published with Federal Agency Partners at the CDC and NCI, unfunded Affiliate members and CPCRN Scholars from outside institutions. CPCRN members have collaborated with investigators on six continents, most frequently with international colleagues from Canada, the Netherlands, the United Kingdom, Australia, Mexico, and Italy (Fig. 3). As an example, in 2019, members of CPCRN’s Organizational Theory for Implementation Science Workgroup collaborated with an investigator from Linköping University in Sweden on a workgroup publication titled “Advancing the Use of Organization Theory in Implementation Science” [31]. In 2022, investigators from CPCRN Collaborating Centers at the University of Arizona and the University of North Carolina at Chapel Hill collaborated with investigators from the National Institute of Perinatology in Mexico and Queen’s University in Canada on a systematic review and meta-analysis on factors associated with cancer treatment delay [32]. They have also developed effective and enduring partnerships with community organizations and healthcare partners.

**Table 3:** CPCRN Collaborating Centers (2002–2024)

CPCRN1: 2002–2004	CPCRN2: 2004–2009	CPCRN 3: 2009–2014	CPCRN4: 2014–2019	CPCRN5: 2019–2024
University of Washington	University of Washington	University of Washington	University of Washington	University of Washington
University of South Carolina		University of South Carolina	University of South Carolina	University of South Carolina
University of Kentucky—West Virginia University^a^			University of Kentucky	
Harvard University	Harvard University	Harvard University		
University of Texas Health Science Center at Houston	University of Texas Health Science Center at Houston	University of Texas Health Science Center at Houston		
	University of North Carolina at Chapel Hill (*Coordinating Center & Collaborating Center*)	University of North Carolina at Chapel Hill (*Coordinating Center & Collaborating Center*)	University of North Carolina at Chapel Hill (*Coordinating Center & Collaborating Center*)	University of North Carolina at Chapel Hill (*Coordinating Center & Collaborating Center*)
	Emory University	Emory University		Emory University
	University of California, Los Angeles	University of California, Los Angeles		
	Morehouse School of Medicine			
	St. Louis University			
		Colorado School of Public Health		Colorado School of Public Health
		Washington University		
		Texas A&M University		
			University of Iowa	University of Iowa
			Case Western Reserve University	
			Oregon Health & Science University	
			University of Pennsylvania	
				New York University—City University of New York
				University of Arizona

^a^The University of Kentucky (UK) and West Virginia University (WVU) PRCs teamed up in CPCRN1 to collaborate on the development of the CPCRN Coordinating Center, subsequently introduced as a permanent facet of Network structure beginning in CPCRN2 (2004). UK rejoined the Network in CPCRN4 as an independent PRC, as WVU’s involvement was discontinued

**Fig. 4:**
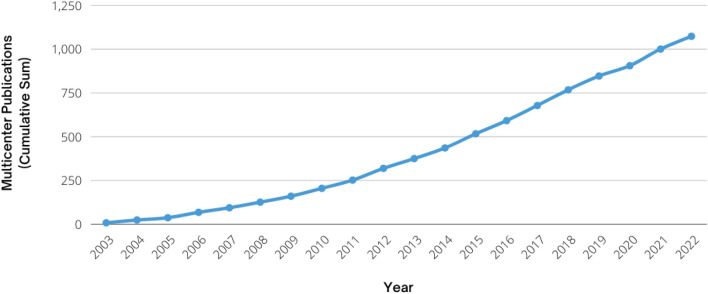
Cumulative sum of the CPCRN multicenter publications over time (2003–2022) (Publication data are not available for the 2002 funding year, as the first CPCRN cycle effectively began in October 2002, and 3 months’ time would not have been sufficient to yield cross-center publications)

Multicenter publications are one indicator used to gauge the level of collaborative impact within the Network. Across the 20 years of CPCRN (2003–2022), CPCRN members have produced 22,781 total publications (journal articles, reviews, conference papers, and book chapters), of which 1,074 are multicenter publications with at least two authors from different CPCRN institutions. In 2003, the Network produced eight multicenter publications; in contrast, by 2021, the last full year for which data are available, the number of multicenter publications produced in a single year grew to almost 100 publications (Fig. 4). Meaningful contributions to the field include advancing knowledge in specific content areas (such as interventions to improve HPV vaccination, cancer screening, treatment and survivorship) as well as contributions to the larger field of implementation science, including the development and use of the Putting Public Health Evidence into Action training curriculum for capacity building, contributions to the application of relevant theories and frameworks in implementation science, and focusing on integration of participatory community engagement, application of systems science methods, and economic modeling in implementation science.

**Fig. 5:**
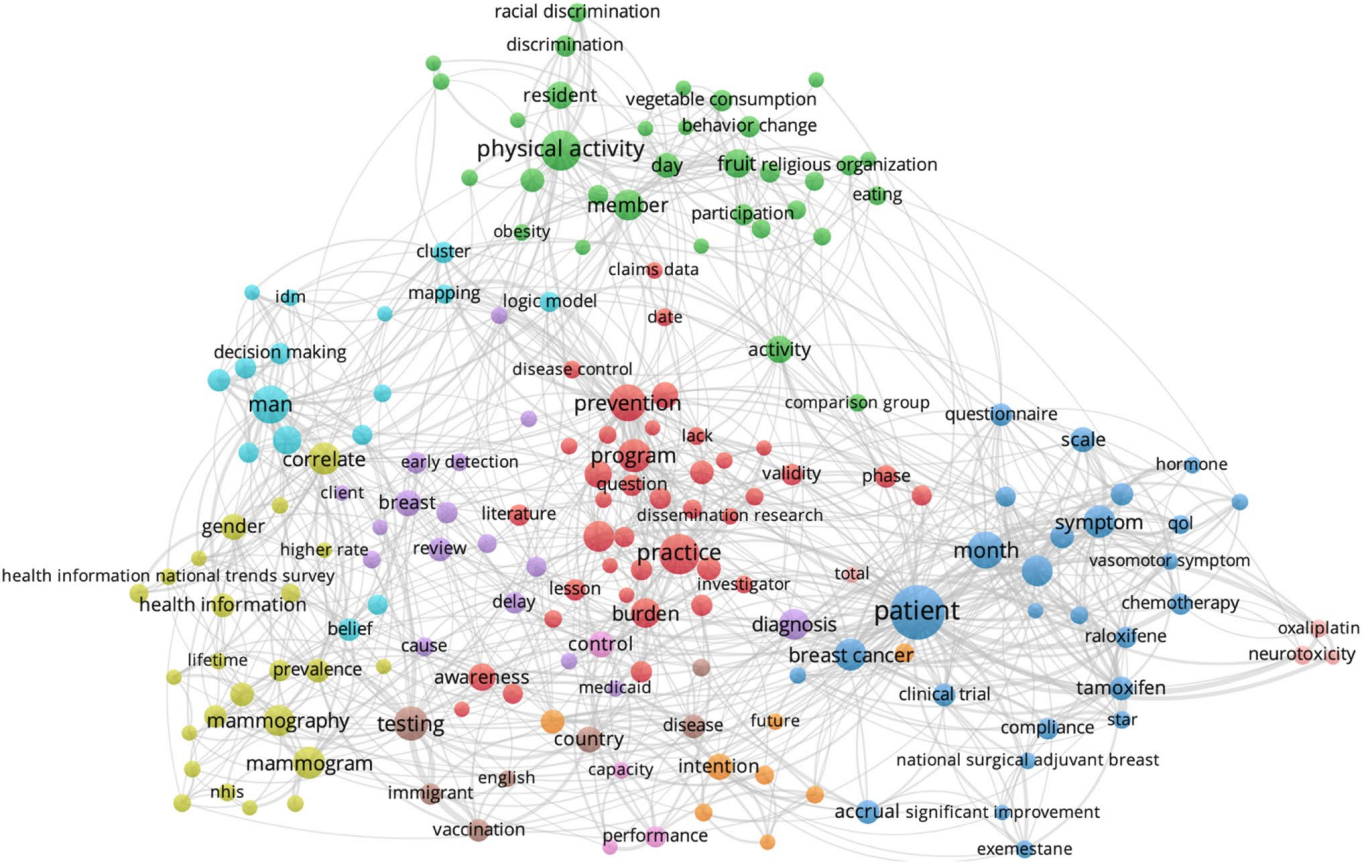
Key term clusters from the CPCRN multicenter publication titles and abstracts, 2005–2009 (Publication data are reported for unique funding cycle periods to illustrate key content area themes from analysis of MeSH terms and keywords within publications during that period)

**Fig. 6:**
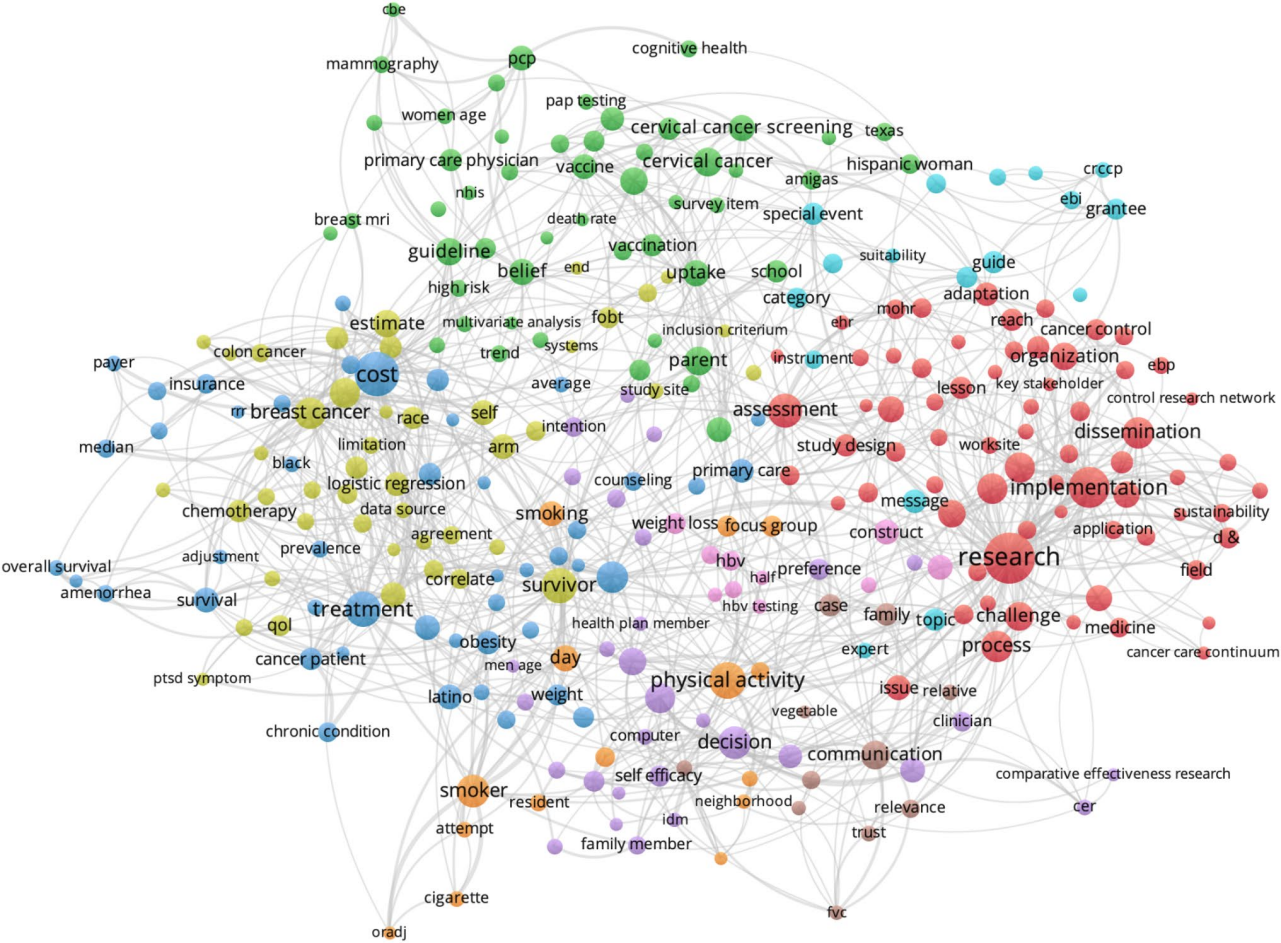
Key term clusters from the CPCRN multicenter publication titles and abstracts, 2010–2014 (Publication data are reported for unique funding cycle periods to illustrate key content area themes from analysis of MeSH terms and keywords within publications during that period)

**Fig. 7:**
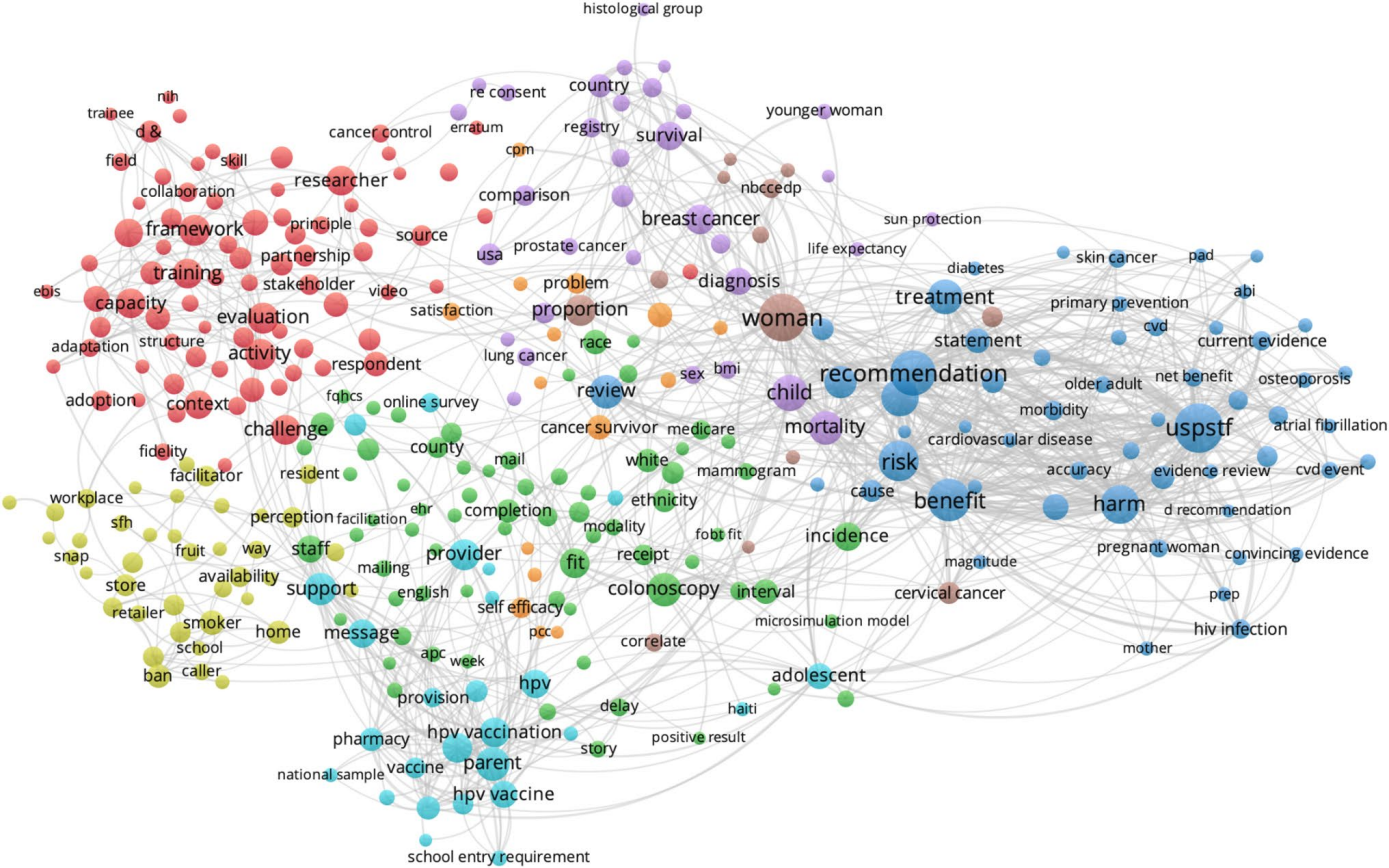
Key term clusters from the CPCRN multicenter publication titles and abstracts, 2015–2019 (Publication data are reported for unique funding cycle periods to illustrate key content area themes from analysis of MeSH terms and keywords within publications during that period)

**Fig. 8:**
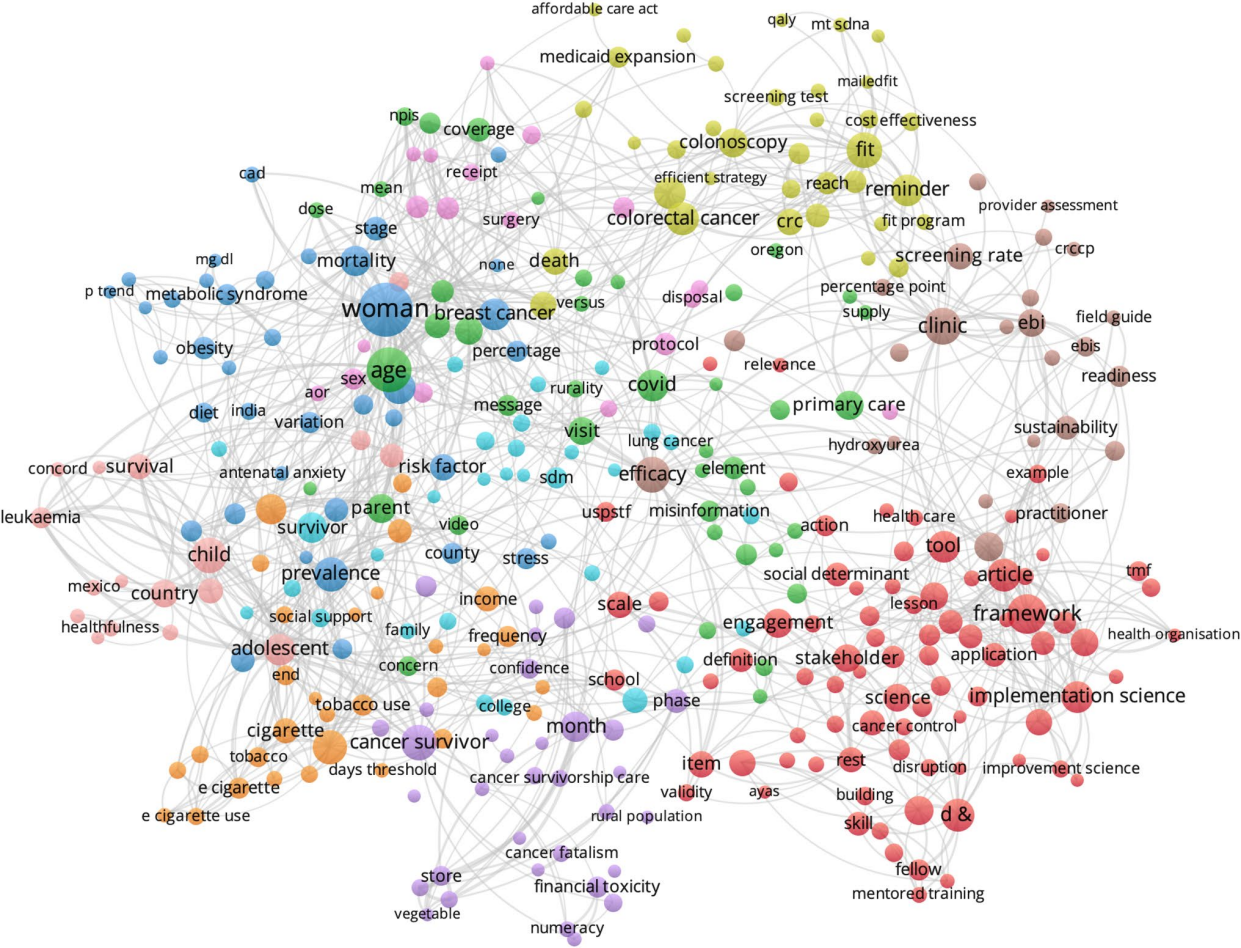
Key term clusters from the CPCRN multicenter publication titles and abstracts, 2020–2022 (Publication data are reported for unique funding cycle periods to illustrate key content area themes from analysis of MeSH terms and keywords within publications during that period)

Cluster analysis of key terms from titles and abstracts of CPCRN multicenter publications provide some indication of how the topic emphasis of CPCRN research has shifted over time as members’ or centers’ expertise or interests have evolved (Figs. 5, 6, 7, 8). During the 2005–2009 cycle, major areas of focus included physical activity and health behaviors, along with mammography and health information clusters. In 2010–2014, clusters emerged around cost, cervical cancer and HPV vaccination, cancer survivorship, and implementation. From 2015 to 2019, USPSTF recommendations, frameworks, community engagement, and colorectal cancer screening were key clusters within the Network. Most recently, from 2020 to 2022, the Network has developed an enhanced focus on financial toxicity/financial hardship, social determinants of health, and structural racism. Since the beginning, CPCRN has consistently focused on dissemination and implementation of evidence-based cancer prevention and control interventions and community outreach and engagement to enhance cancer health equity.

A few examples of community partnered projects are: (1) the University of Arizona establishing an academic-community partnership with Mariposa Community Health Center (MCHC) to improve the health and reduce morbidity among border-dwelling Hispanic cancer survivors; (2) the University of Iowa College of Public Health launching the Health Equity Advancement Lab (HEAL) in 2012 to support researchers and non-academic community partners interested in tackling persistent health inequities through a community-based participatory research approach; and (3) the University of North Carolina partnering with four federally qualified health centers and health departments to increase implementation of colorectal cancer screening, lung cancer screening, HPV vaccination, and HPV/Pap co-testing.

#### Collaborative grants

**Fig. 9:**
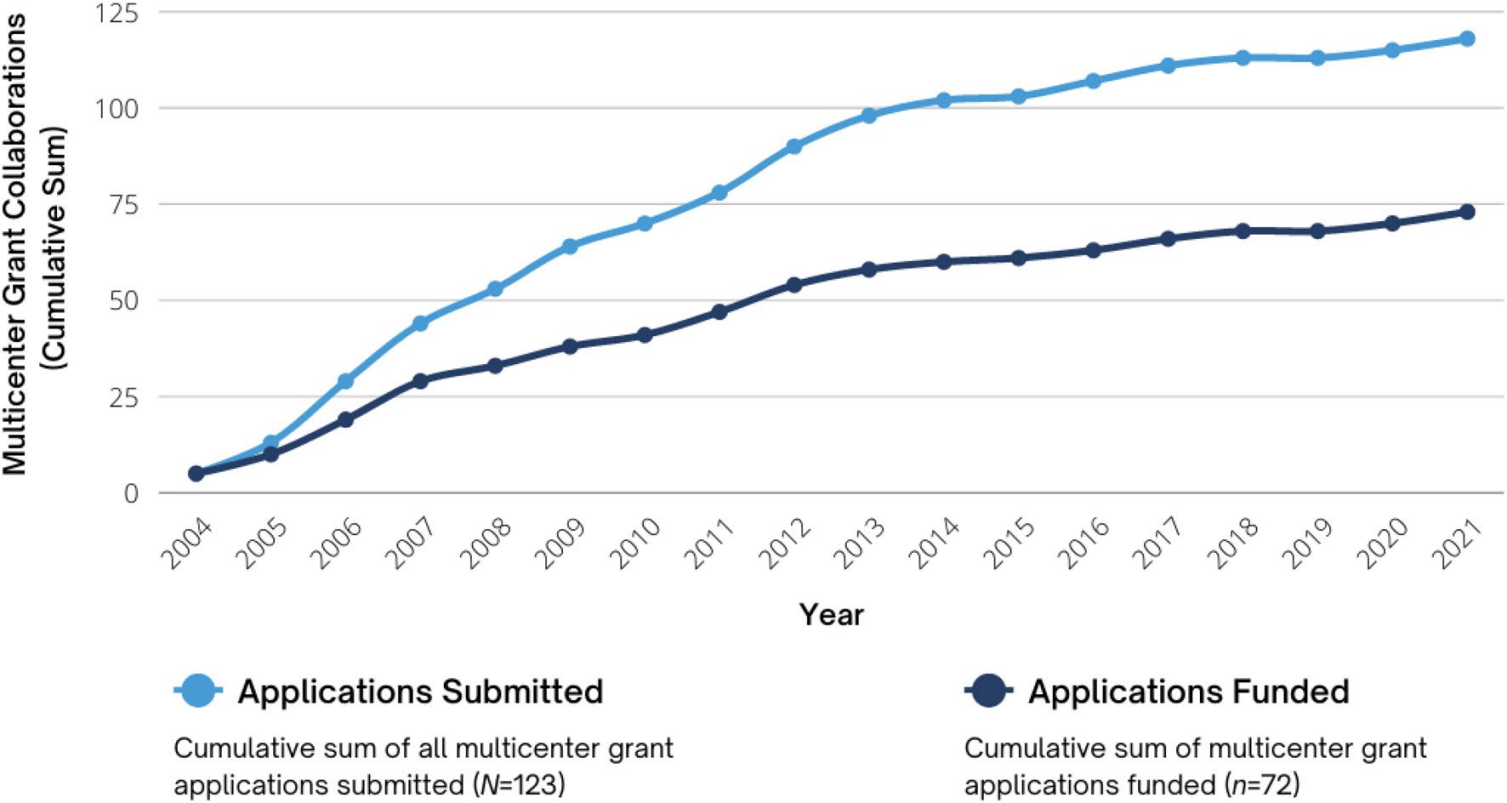
Cumulative sum of multicenter grant collaborations over time (2004–2021) (Grants data were not collected in 2002–2003, nor have they been collected for the 2022 funding year to date)

CPCRN began collecting grants data in 2004. Since that time, CPCRN members have received a total of 682 grants in support of research efforts to advance the adoption and implementation of evidence-based cancer prevention and control. Of the 123 multicenter collaborative grant applications submitted since 2004, 72 (~ 59%) have been funded, resulting in over $77.1 million in funding support for collaborative CPCRN-related research (Fig. 9). For example, investigators from the University of North Carolina at Chapel Hill, University of Kentucky, and Oregon Health and Sciences University were awarded NCI Cancer Moonshot grants focused on Accelerating Colorectal Cancer Screening and Follow-up Through Implementation Science (ACCSIS) and regularly consult with and publish together on colorectal cancer screening topics. All leveraged their involvement in CPCRN to obtain these grants.

### Qualitative

A total of 22 key informants were interviewed over a 3-month window in July–September 2022, representing a diverse group of CPCRN members whose duration of involvement in the Network ranged from three to all 20 years. PIs, PDs, Federal Agency Partners, and community partners were included. In-depth content analyses of these data revealed eight prominent themes of the Network that have significantly contributed to the success and impact of CPCRN: Multidisciplinary expertise; Collaborative culture; Supportive funding; Network structures and policies; Professional and leadership development; Community orientation; Adaptability; and Ongoing learning and improvement. These eight themes can be grouped into three overarching meta-themes: (1) inclusion of *people* representing diverse content-area interests, multidisciplinary perspectives, and geographic and socioeconomic contexts; (2) dedicated, centralized *structures and processes* designed to promote healthy collaboration and the ability to meaningfully evaluate those efforts; and (3) focused attention around *strategic adaptation* in response to rapidly-changing evidence and to meet the dynamic, contextually specific needs and interests of community partners.

Each of the eight themes and corresponding sub themes are summarized below. A comprehensive breakdown of the full codebook, along with de-identified, illustrative quotations drawn from the discussions to further describe the codes in context, is described in Table 4.

**Table 4:** Primary themes, subthemes, and illustrative quotations about the special sauce of the CPCRN

Themes & subthemes	Definitions & illustrative quotations
1. Multidisciplinary expertise	A rich and diverse group of experts comprise the Network, offering an extensive range of backgrounds, experience, and subject matter (content, methods, and setting) expertise
	“Having a network of colleagues that you can refer other people to or that you can go to for as consultants… I just think you have this network of experts that you can tap into for so many things” “…diversity of the different researchers in terms of where they are [located], what populations they are focused on, what areas they have expertise in, as well as you know, diversity in areas of work across differences races, ethnicities, rural [vs.] urban [populations], etc”
2. Collaborative culture	Nature by which Network members engage with one another within their own centers and across the Network, and the types of relationships that stem from those engagements
2.1 Shared motivations and values	“It’s just this really supportive [group who is] really dedicated to important issues like training the next generation, working with community members, health equity and moving evidence-based research to practice” “There’s been a lot of sharing of best practices of the science, of grants management, and, you know, how folks do [other] stuff that can and have just benefitted the entire Network”
2.2 Longevity	“CPCRN, in my mind, is very productive. Some of that is the longevity, it’s the history, and it’s understanding the processes and the types of work that can be done. And also, the trust among the folks that are engaged, the willingness to take risks, and to share what you’re doing so that the sum of this Network is actually more than just the individually-funded parts” “CPCRN had funded several of these Collaborating Centers over the course of multiple [funding cycles], and so that stability, I think, really allowed this Network to cumulatively and exponentially be productive over time”
2.3 Collegiality	“It’s the people… having a group–collegial, smart, dedicated, passionate, committed, you know, pick your adjective–who just want to do the right thing”
3. Supportive funding	Monetary awards acquired by academic cancers and individual investigators to support scientific research efforts related to cancer prevention and control
3.1 Funding amount ($)	“[CPCRN has] had dedicated funding for over 20 years” “…CPCRN and the dollars from [Federal Agency] were kind of the vehicle for [us] to be able to work together” “We just have to be reasonable with expectations and what we’re all able to do with the funding that we have” “The fact that there’s actually money behind cross-center collaboration, I think, is huge”
3.2 Funding expectations	“It’s even in the funding, it’s baked in that half of your funding needs to go to cross-center projects and [the other] half of your funding goes to your own [core] center projects” “I think the funding piece is huge, the expectation being that everyone’s going to collaborate and there’s money behind that collaboration” “We made it a funding requirement that at least 50% of the resources that the Collaborating Centers received had to be spent on collaborations with other investigators [from other Centers]”
3.3 Funding mechanism (grant type)	“I’ve never worked with other universities on projects like this, except within [CPCRN]. That’s the Network magic. That’s what [the funding] supports. Otherwise, what you have is, like, a multicenter trial, or, you know, there aren’t other funding mechanisms that support that kind of collaboration and networking”
3.4 Federal Agency Partner accessibility	“Having funders that really help to drive the Network and be really engaged at the table, expressing their satisfaction or dissatisfaction with productivity or progress, was really helpful” “Funders, they’re collaborators, they’re working with the PIs and faculty and we know who they are and can reach out if we have questions”
3.5 Additional grant applications and awards	“There’s been some calculation on return on investment… CPCRN dollars have been leveraged to write more grants and bring in more funding to the investigators and their [Collaborating Center] teams” “The money that is funded in the CPCRN is designed to bring [Collaborating] Centers together, to get them to work together to move the field forward” “I think that there’s been some great grant applications that have come out of [CPCRN]”
4. Network structure and policies	The fundamental organizational breakdown, processes, and functions that characterize the CPCRN and its members, and by which the Network operates
4.1 Coordinating center	“I definitely think the Coordinating Center has been a great facilitator of the work [of the Network], and having that structure in place, I think, has played a huge role in [the Network] being as productive as it [has been]”
4.2 Principal investigators (PIs)	“I didn’t just all of a sudden happen where, ‘Boom!’ and I appeared. (laughs) I’m, you know, part of the Network, I gradually was pulled in because the PIs and the PDs pulled me in, supported me, funded my projects, invited me into leadership roles, and incorporated me”
4.3 Project directors (PDs)	“…not only is leadership at the PI level, but talking to the Project Directors, they’re different group members that also work in individual [Network] Centers, as well as [on] cross-center [projects], and help us during our meetings. Having this interaction, I see that their commitment flows out to everyone. It trickles down and really flows in[to] every layer of the research team”
4.4 Steering committee (SC)	“…and on Steering Committee calls, [leadership] oftentimes will open up the floor for feedback and suggestions, and it’s a very collaborative process for shared decision-making within the Network”
4.5 Chair/co-chair roles	“…the way the Coordinating Center has structured the way that PIs cycle in-and-out of the leadership role, the way [they] have the co-chair calls every month, those mechanisms are put in place to facilitate [the exchange of new ideas and ensuring that all voices are heard]. So, I feel like the infrastructure of the Network and the Coordinating Center support that ongoing learning that occurs amongst all of the parties”
4.6 Center-specific core projects & cross-center workgroups/interest groups	“There are other networks where there [are] a lot of centers funded and everyone’s just doing their own thing. But, I think there is no point in doing this [kind of funding] unless there’s going to be some cross-center work… it’s led to, I think, multicenter success [for CPCRN]”
4.7 Network leaders	“…I think [one thing that has allowed the Network to be meaningfully impactful is] our leadership’s ability to see beyond one research project, and to think about systems; to think about, you know, policies; to think about larger change”
4.8 Affiliate membership	“You have people that aren’t necessarily funded, but [they] do see a benefit in being engaged with this Network” “[CPCRN is] a great network … of, you know, the funded [Collaborating] Centers, and then also the way that we’ve established the Affiliate [membership] and the way that we’ve still collaborated with people who used to be funded in the CPCRN and invited all kinds of new people to the table as well”
4.9 Progress reporting	“I would commend the CPCRN for embracing that [CDC] Science Impact Framework and the way that [the Coordinating Center has] used that to describe [the Network’s] accomplishments”
5. Professional and leadership development	Noteworthy personal and professional achievements earned, opportunities grasped, milestones reached, and evolution into leaders of respective fields, attributable in part or in full to the CPCRN participation
5.1 CPCRN scholars program	“When I was very junior, people were mindful of career development and trying to create opportunities for folks in the Network earlier in their careers… that’s where I’ve seen amazing, continued progression and growth, is just that general mindfulness to what we have today, where there’s a whole [CPCRN] Workgroup focused on scholarship, mentorship, and development”
5.2 Mentorship	“Truly, I think the benefits and one of the strengths of the Network is the commitment towards mentorship of others. And I deliberately say others, broadly, because I think that I’m not referring to just junior investigators, but research staff as well. I see mentorship, and I’ve heard about mentorship along the investigator spectrum, which has been really encouraging to hear about”
5.3 Collaboration and partnerships beyond the network	“These [CPCRN] collaborations extend well beyond the Network… I’ve looked for experts from within the CPCRN multiple times, because, you know, a lot of it is just the collaboration and the networking”
6. Community orientation	Recognizing and addressing social and environmental determinants of health at the community level through research efforts, often in collaboration with the communities themselves, to educate, build capacity, and improve public health outcomes
6.1 Community-based participatory research (CBPR)	“I think the Network has been really mindful about community partner needs and serving them, not using them” “I’ve worked with a lot of very empowered community partners [in the Network] who are very, very comfortable advocating for themselves and their constituents. So, that’s been a blessing”
6.2 Capacity building activities	“The value [in community engagement] is in training and building that capacity that stays within the community, so that [community partners] can go well beyond the funding we provide them with and maintain some of the work that we’ve done” “One of the things that starts to happen as you build some of that capacity in the community is, suddenly, [community members] become a very valuable partner to a researcher; before that partnership, the walls were too high. There’s a number of those [CPCRN-led/-involved efforts] that have been very successful in that way”
6.3 Dissemination	“…[CPCRN] Centers have great results…and better outcomes and you see greater impact from the Network, you see these products like a training or a toolkit or something that can be disseminated and scaled-up so that other folks don’t have to reinvent the wheel. I think those are the big wins that we love to see”
6.4 Evidence-based interventions (EBIs)	“We are able to provide trainings to our community grantees to help them understand, identify, and adapt evidence-based interventions for implementation in their communities. And so, that was a really excellent link that we made, and we were even able to take that training and adapt it for [U.S. state]”
7. Adaptability	Degree to which the CPCRN recognizes and is receptive to need for change, makes informed, joint decisions about the best course of action, executes change as intended, and modifies/sustains the change over time to align with whomever/whatever is driving it
7.1 Changing social & political environment	“We don’t need to just be doing work to write papers. We need to be doing work to change how things are done in the field in a way that aligns with the greater social context. And that’s what I believe implementation is about: come up with an idea, then think, ‘How do you best implement that idea in the real world?’”
7.2 Changing priorities & community needs in the field	“We did a market survey of 2,000 [local health department] employees… they were like, ‘mental health and work-life balance, that’s what we care about.’ And that doesn’t mean we can’t continue to have current disease components in our intervention, but it says to me that we need to adapt [our project] now to include those topics that matter so much… Because our [community] partners are spot on in terms of what people are hungry for and need the most in this moment”
7.3 Changing/new expectations of the network	“With how fast circumstances can and have changed, for everyone, we see that the need and priorities shift quickly. So, [we] just want to make sure that we can share if we feel like there’s a better process or approach that either the Coordinating Center or the Network, structurally, could kind of adapt to and work toward that”
7.4 Funding cycle transitions	“It takes a while to get things going, especially with a new [funding] cycle. And when you have new funded [Collaborating Centers] … we were all learning about each other again” “It’s not surprising that a Network like this that changes every 5 years through competitive renewals, the partners at the table are changing, so you have to recreate or re-establish some of those relationships”
8. Attention to ongoing learning and improvement	Constructive criticisms of the Network and suggestions for adapting existing/adopting new practices to ensure that the CPCRN maintains forward momentum and continues to evolve and have meaningful impact in the years to come
8.1 Prioritization of multicenter (workgroup/interest group) projects	“There’s that challenge of trade-off between the depth of the work that we can do versus the breadth. We’re doing a lot of little things in different areas, because there’s a lot of different interests… but what can realistically be done once money starts to get divvied up by each [Collaborating] Center and only fifty percent of the dollars can go to these cross-cutting projects?” “It’s okay to sunset a Workgroup. And it’s okay to have smaller, short-term Workgroups that don’t necessarily involve every [Collaborating] Center. We can do something very specific and time-bound… and I hope that’s still true, that we do still have some large Workgroups with large scopes of work, but we also just have some small, more organic groups that come and go”
8.2 Emphasis on equity and/or disparities-reduction	“In the last several years, there’s been more of an explicit call-out around equity as the goal. And I would say, before that, ‘health disparities’ research was the catch-all term. But we really need to continue rethinking things, as equity is about trying to match what one needs and making sure that people have equal opportunity, and ultimately, do as well as they possibly can. We’re really trying to level the playing field, which, at least [to me], seems to be what equity is about”

#### Theme 1: multidisciplinary expertise

The breadth of expertise that comprises the Network has become increasingly diverse over time. The current composition of members reflects an extensive range of expertise across content areas (e.g., cancer screening; tobacco cessation; survivorship), disciplines (e.g., epidemiology; economics; health services research, behavioral science) and partnerships/settings (e.g., federally qualified health centers; faith-based settings) across a variety of organizations and representing broad geographic regions and diverse institutions, including minority serving institutions, NCI-designated cancer centers, and more.

#### Theme 2: collaborative culture

The nature by which CPCRN members engage with one another within their own Centers and across the Network, and the types of relationships that stem from those engagements, are seen as inherently collaborative, welcoming, and accessible. Key subthemes related to collaborative culture include: shared motivations and values; longevity of the Network; culture of collegiality; democratic decision processes; and CPCRN being a network of friends and “family.”

#### Theme 3: supportive funding

The sustained and flexible funding from CDC available to Collaborating Centers to support CPCRN efforts, according to key informant interviewees, has been crucial to maintaining and growing operations over time and has allowed academic centers and individual investigators to support early career scholars, community partners, and bold scientific research efforts directly in ways that may not have been possible otherwise. The flexibility of CPCRN funding relates to its focus on building a collaborative research infrastructure where cross-center projects can emerge organically over time as researcher and partner interests align. As a result, the Network can be nimbler to quickly pivot to pursue new topics of shared interest. Key subthemes related to funding include: award amount (e.g., having enough funds to sustain community partnership engagement and to support project staff and co-investigators’ time participating in cross-center activities); allocation expectations (i.e., all centers are expected to expend more than half of their funds in cross-center collaborative activities); funding mechanism and its role in Federal Agency Partner accessibility (i.e., CDC and NCI partners are active scientific collaborators and help set strategic vision for the Network); and additional grant applications and awards pursued by Network members (e.g., being part of CPCRN has enabled investigators to leverage their involvement in CPCRN and associated preliminary data, resources and connections to secure additional external funding to support larger or next-step research projects).

#### Theme 4: network structure and policies

The organizational structure, operating processes and practices, and day-to-day functioning of the CPCRN reflect an intentional shared leadership model bolstered by collaborative guidance documents and procedures developed by the Coordinating Center with input from the Steering Committee. The structure of the CPCRN–consisting of eight funded Collaborating Centers, one funded Coordinating Center, Federal Agency Partners from the CDC and the NCI, an active Steering Committee that meets monthly, and multiple priority Workgroups and Interest Groups–has enhanced its capability to conduct work efficiently and effectively. The CPCRN Steering Committee consists of the Coordinating Center PI, PIs from all funded Collaborating Centers, Federal Agency Partners from the CDC and NCI, and all Workgroup and Interest Group leaders. Each year, two Collaborating Center PIs serve as Steering Committee co-chairs. Collaborating Center PIs work closely with their local communities and bring feedback from their community partners to the Steering Committee. Key subthemes related to Network structure include: its engaged key personnel consisting of PIs, PDs, Co-Investigators (Co-I), Federal Agency Partners, Affiliates, intra-Network entities and community partners; the structure of Center-specific (Core) and multicenter (Workgroup and Interest Group) Projects; the development of strong guidance documents written, maintained, and updated over time; strength of Network leadership; development of an Affiliate membership process and inclusive orientation; use of a robust progress reporting structure; and strong Network communications structures.

#### Theme 5: professional and leadership development

Network members often attribute their noteworthy professional achievements attained, opportunities seized, milestones reached, and their evolution into leaders in the Network and in their respective fields, in large part, to their involvement in the CPCRN. Key subthemes related to professional and leadership development include: a strong commitment to mentorship across the Network; member and community partner engagement and collaborations beyond the Network; and the development and growth of the CPCRN Scholars Program to formalize training activities for the next generation.

#### Theme 6: community orientation

The CPCRN has always been community-connected-oriented—a network of networks strengthened by community advisors’ input within each Collaborating Center—with an explicit focus on recognizing and addressing social and environmental determinants of health at the community level, building capacity for knowledge translation and adaptation in diverse settings, and improving public health outcomes, especially for the most marginalized communities. Key subthemes related to community orientation include: emphasis on community-based participatory research and engaged research; focus on capacity-building activities; active dissemination of research products; and production of evidence-based interventions.

#### Theme 7: adaptability

Adaptability reflects the degree to which the CPCRN recognizes and is receptive to the need for change, makes informed, joint decisions about the best course of action, and sustains changes over time, as needed, to align with the forces driving for change and in the best interests of the Network. An open, continuous-learning mindset allows the CPCRN to adapt more quickly to new challenges (e.g., providing support for asynchronous Workgroup activities and flexibility during the COVID-19 pandemic) and to be responsive to opportunities for synergy (e.g., facilitating cross-Collaborating Center communications when Workgroups share interest in similar topics). Key subthemes related to adaptability include responding to: the social and political environment; priorities and needs in the field; expectations of the funders and Network members; and the CPCRN funding cycle fluctuations and transitions.

#### Theme 8: attention to ongoing learning and improvement

Being attentive to lessons learned and being open to, and responding directly, to critical feedback are core principles that guide the CPCRN functions. This theme reflects lessons learned through involvement in the Network, as well as constructive criticisms, identification of opportunities for improvement, and suggestions for modifying existing practices/adopting new ones to ensure that the Network maintains forward momentum and continues to grow and have impact in years to come. Opportunities for improvement noted in the interviews were: increasing the representation of underrepresented investigators in the CPCRN; continuing to embed health equity principles in all network functions; identifying more ways to support the rapid growth in numbers of unfunded Affiliates and scholars; creating more mechanisms for community partner input; and ensuring that the network can sustain its work and momentum across funding cycle transitions.

#### Policies and procedures documents review

When assessed altogether, the policies and procedures documents, Network progress reporting and Workgroup charter templates, and other miscellaneous resources were collectively effective in painting a complete and comprehensible picture of the CPCRN document infrastructure, including the breakdown of roles, intra-Network dynamics, and functions and processes integral to, and increasingly characteristic of the CPCRN. The CPCRN Coordinating Center has developed guidance resources, communication infrastructures, and efficient processes for supporting integration and logistics among Network members and continues to create new materials to improve productivity, visibility, and impact as needed. Network structures and processes have helped clarify roles, functions and expectations of the CPCRN members. This includes establishing productivity norms, deliverables, timelines, and metrics up front when orienting new Collaborating Centers and Affiliate members, chartering a Workgroup/Interest Group, and monitoring leadership and dynamics at regular intervals. Among other noteworthy takeaways from the documents analysis was a recurring emphasis—explicitly stated or clearly illustrated—regarding the critical importance of promoting new and bolstering existing multidisciplinary collaborations within and across Network centers (to the extent that they are still productive and meaningful investments of time in resources for all parties involved) (Table 5).

**Table 5:** The CPCRN guidance documents

The CPCRN policies & procedures
Elements of a vision for the CPCRN
The CPCRN logic model
Expectations for investigators funded through the CPCRN
The CPCRN progress reporting: overview of reporting obligations
Steering committee roles and responsibilities
Coordinating center roles and responsibilities
The CPCRN workgroup formation process
The CPCRN workgroup best practices
Guidelines for collaboration
Funding Acknowledgement Policy
CDC Publications Clearance Policy
The CPCRN Communications Plan
Affiliate Member Policy
The CPCRN Strategic Plan
CDC science impact framework—key indicators
Workgroup progress report template
The CPCRN workgroup formation concept paper template
The CPCRN workgroup charter template
Affiliate member application form
Other the CPCRN documents
Progress reporting guidance
The CPCRN style guide—logos and color palette
The CPCRN templates—slides, data briefs, and report covers

The CPCRN Coordinating Center has tracked self-reported disciplinary strengths and methods expertise of its members for the past two cycles of funding. These data have been used to create network cluster diagrams in Kumu, which are available on our website for investigators, Federal Agency Partners and others to use to help identify collaborators, subject matter experts, or to understand network members’ research expertise (https://www.cpcrn.org/about-areas-of-expertise). Current cycle data indicate that CPCRN5 (2019–2024) members represent diverse disciplines, including epidemiology, health behavior, health services research, health economics, nutrition, nursing, medicine, and pharmacy and possess a broad range of research methods expertise, including program evaluation, survey development, simulation and systems science, community engagement and community-based participatory research (CBPR), capacity building, training and training evaluation, providing technical assistance, economic evaluation, health and risk communication, dissemination, implementation science, de-implementation, social network analyses, intervention development and testing, measures/measures development, qualitative methods/configurational comparative methods, large database analytics, systematic review/meta-analysis, human-centered design/design thinking/innovation techniques, comparative effectiveness, mHealth/eHealth, and spatial analysis/GIS and mapping.

## Discussion

The CPCRN is a longstanding multicenter collaborative initiative that is unique in its organizational structure, productivity, and longevity across its 20-year history. Its members continue to innovate, develop, evaluate, implement, and scale-up cancer prevention and control evidence-based approaches with their local, state, national, and international partners, influencing everything from local clinic practices and state cancer plans to national organizations’ practices and policies. The current mixed methods analysis employing bibliometric techniques and qualitative methods illustrates the broad scope of Network collaboration. Our analysis not only highlighted the substantial growth in multidisciplinary and multifocal collaborative research over time, but also clarified the key drivers that have facilitated and strengthened Network cohesion and collaboration over time—i.e., the “special sauce” of the CPCRN—its tremendous *people* power, representing diverse content-area interests, multidisciplinary perspectives, and geographic and socioeconomic contexts; dedicated *centralized structures and processes* to enable and evaluate collaboration; and *willingness to adapt* over time.

Adaptation and change can be challenging for loosely connected academic networks like the CPCRN that comprise different institutions and individuals. The Competing Values Framework helps to clarify how a network like the CPCRN may be able to succeed despite continuous change resulting from turnover in membership and ongoing (re)prioritization of activities [33–35]. Specifically, the CPCRN must balance both an internal focus on its membership and external focus on funder priorities, scientific and sociopolitical movements, and externally driven resource constraints. The Network also must balance a focus on flexibility (in response to change and shifting priorities) and control (offering stability and collaborative structure to its membership). In addition, the Network must balance a focus on task completion (productivity) with a focus on people (human development). Our data suggest that the organizational culture of the CPCRN is more team-oriented and entrepreneurial than hierarchical, for example, but that rational prioritization of tasks, given competing demands, is essential. It is the dynamic balancing of these competing values or orientations that makes the CPCRN work well over a 20 year period. The Competing Values Framework can also be used going forward to inform future network strategic planning and prioritization in light of ongoing challenges to balance multiple competing demands.

Large, complex research networks with competing values and demands need effective coordination, communication, and facilitation to ensure that the work of investigators, collaborating partners, and Affiliate members occurs smoothly, is completed in a timely manner, and meets Network goals, which is facilitated by well-organized structures and processes centralized within the Coordinating Center. The introduction of new Collaborating Center institutions over time—a process that occurs in 5-year funding cycles with the PRC Program and engagement of new community, and clinical partners and individual investigators on an ongoing basis—can bring a fresh perspective and different strengths to existing the CPCRN partnerships. On the other hand, turnover can sometimes introduce uncertainty and confusion since new members are not as familiar with the culture and norms of the Network. The Policies and Procedures guidance document and other resources help to orient new members to the Network and engage them quickly in Network activities. Because multicenter collaboration is the *raison d’etre* for the Network, the CPCRN employs an inclusive engagement process to invite and support new collaborative ideas and opportunities, aided and vetted by the Steering Committee, which evaluates whether new collaborative ideas reflect the objectives and priorities of the CPCRN, as well as the expertise and skills of participating member institutions and individual investigators and Affiliate members. This process allows new Workgroups to emerge organically, to adapt strategically, and then dissolve, if appropriate, when their work is complete.

This shared ownership and decision-making process was viewed by key informants as helpful in keeping members connected and productive over time. The Network has clearly grown in terms of its membership and publication productivity from its 10-year anniversary bibliometric analysis—249 collaborative the CPCRN publications were reported out of 6,534 total articles among 309 unique the CPCRN researchers at 11 centers—to its 20-year anniversary—1,074 collaborative publications were reported out of 22,781 total articles among more than 600 unique the CPCRN researchers at 17 centers [13]. In addition, its grant record is remarkable with nearly 60% of submitted grants being funded. Beyond publications and grants, interviewees noted the CPCRN members’ roles serving as subject matter experts, influencing guidelines and policies, building community capacity for cancer control, and contributing to important advances in the field [13, 14, 18, 19, 31, 34–37]. the CPCRN places strong value and relies heavily upon shared leadership, consensus-driven decision making, and mutual accountability—principles that reflect an appreciation of and commitment to harnessing the CPCRN’s “people power.” As evidenced by the key informant interviews, not only do the CPCRN members believe in the mission and vision of the CPCRN scientifically, but many also feel deeply connected to the Network that supported their professional growth, expanded their personal and professional connections, and extended leadership and scholarly opportunities to them that they otherwise might not have had. The CPCRN’s more recent formation of the CPCRN Scholars Program and development of the CPCRN Health Equity Principles in the current funding cycle further underscore and formalize longstanding member interests in supporting the next generation of cancer prevention and control researchers, particularly those focused on cancer health equity and those from underrepresented backgrounds [38, 39]. The Network’s Health Equity Principles are reflected in ongoing research and engagement activities and guide continued growth in collaborations focused on addressing disparities across geographic boundaries and institutions [39, 40].

Some limitations accompany this work. First, for the bibliometric analysis, we chose to define center-level identifiers as the individual institutions with which each author was affiliated at the time that they entered the Network. Some authors were affiliated with two or more institutions during their respective years of involvement. As such, this decision may have resulted in a small number of publications being incorrectly attributed to authors’ original centers; however, because the focus of the Network is on multicenter collaborations rather than tracking individual authors’ professional trajectories, this was felt to be a reasonable approach. Second, for the interviews, key informants whose involvement was limited to early years of the CPCRN were less likely to respond to our invitations to participate (due to retirements or other reasons), which may have biased our analysis towards the inclusion of more recent observations. Third, it is, of course, possible that respondents who chose to participate in interviews had more positive experiences and/or perceptions of the CPCRN than non-respondents. Similarly, social desirability bias may have also prompted them to be more complimentary when interviewed by our research assistant. In an effort to account for and minimize risk of collecting socially desirable response data, we invited a broad range of the CPCRN members from multiple institutions and roles over time, using a single research assistant interviewer, de-identifying all data, and assuring privacy and confidentiality. As noted in Theme 8, several opportunities for improvement and future work were noted by interviewees, which suggested that interviewees felt comfortable sharing strengths and weaknesses of the CPCRN. The latter have already been incorporated into network strategic planning and prioritization.

## Conclusions

Our analysis offers some important insights to guide other thematic population health-focused research networks, particularly those in cancer prevention and control, seeking to build scientific synergy and promote community-academic collaboration in a rapidly changing environment. Other networks may seek to adopt similar collaborative values, organizational structures and processes to support remote and multicenter cooperation while facing competing and potentially contradictory demands. Our findings suggest that the special sauce of the CPCRN is made up of three key ingredients: people; processes and structure; and adaptability. Investing in and focusing on the professional development and inclusion of *people* from diverse disciplines, backgrounds and geographic settings and sharing the decision making and priority setting with these individuals—invites meaningful input and continued commitment over time. Developing and adapting infrastructure (policies, guidance, expectations) to support collaboration provides the structure to incorporate new members and guide Network functioning and operations. And finally, being willing to embrace change and balance competing values helps ensure responsiveness, relevance, and longevity of the Network over time. These ingredients help to ensure that the CPCRN will have a lasting impact on the science and practice of cancer prevention and control for many years to come.

## Data Availability

The data generated and analyzed during the current study will be made available through UNC Chapel Hill’s the CPCRN Dataverse account.
